# Detection experiments with humans implicate visual predation as a driver of colour polymorphism dynamics in pygmy grasshoppers

**DOI:** 10.1186/1472-6785-13-17

**Published:** 2013-05-02

**Authors:** Einat Karpestam, Sami Merilaita, Anders Forsman

**Affiliations:** 1 Ecology and Evolution in Microbial Model Systems, EEMIS, Department of Biology and Environmental Science, Linnaeus University, Kalmar, SE-391 82, Sweden; 2 Behavioural and Evolutionary Ecology Group, Environmental and Marine Biology, Department of Biosciences, Åbo Akademi University, Turku, FI-20520, Finland

**Keywords:** Biodiversity, Camouflage, Colour polymorphism, Crypsis, Predation, Evolution, *Tetrix subulata*

## Abstract

**Background:**

Animal colour patterns offer good model systems for studies of biodiversity and evolution of local adaptations. An increasingly popular approach to study the role of selection for camouflage for evolutionary trajectories of animal colour patterns is to present images of prey on paper or computer screens to human ‘predators’. Yet, few attempts have been made to confirm that rates of detection by humans can predict patterns of selection and evolutionary modifications of prey colour patterns in nature. In this study, we first analyzed encounters between human ‘predators’ and images of natural black, grey and striped colour morphs of the polymorphic *Tetrix subulata* pygmy grasshoppers presented on background images of unburnt, intermediate or completely burnt natural habitats. Next, we compared detection rates with estimates of capture probabilities and survival of free-ranging grasshoppers, and with estimates of relative morph frequencies in natural populations.

**Results:**

The proportion of grasshoppers that were detected and time to detection depended on both the colour pattern of the prey and on the type of visual background. Grasshoppers were detected more often and faster on unburnt backgrounds than on 50% and 100% burnt backgrounds. Striped prey were detected less often than grey or black prey on unburnt backgrounds; grey prey were detected more often than black or striped prey on 50% burnt backgrounds; and black prey were detected less often than grey prey on 100% burnt backgrounds. Rates of detection mirrored previously reported rates of capture by humans of free-ranging grasshoppers, as well as morph specific survival in the wild. Rates of detection were also correlated with frequencies of striped, black and grey morphs in samples of *T. subulata* from natural populations that occupied the three habitat types used for the detection experiment.

**Conclusions:**

Our findings demonstrate that crypsis is background-dependent, and implicate visual predation as an important driver of evolutionary modifications of colour polymorphism in pygmy grasshoppers. Our study provides the clearest evidence to date that using humans as ‘predators’ in detection experiments may provide reliable information on the protective values of prey colour patterns and of natural selection and microevolution of camouflage in the wild.

## Background

Animal colour patterns may protect against visually oriented predators by impairing detection or recognition of prey, or by reducing willingness to attack [1-3]. The efficacy of (most types of) protective coloration depends on the visual properties of the animal colour pattern relative to properties of the visual background where the animals live, and this offers excellent opportunities for investigations of evolution of local adaptations [4-6], evolutionary shifts in response to environmental changes e.g., [7-10], and for studies of factors that influence the maintenance and dynamics of polymorphisms e.g., [7,11].

Species that are polymorphic for colour pattern are particularly useful as model systems in this respect, since their discrete variation facilitates comparisons and analyses of morph frequency shifts in patchy or changing environments, as, for instance, in the case of the mangrove snail *Littorina* sp. [12], the crab spider *Xysticus sabulosus*[13], the marine crustacean *Idotea baltica*[14], the walking stick *Timema cristinae*[15,16], and the pygmy grasshoppers *Tetrix japonica*[17] and *T. subulata*[7]. In all these species, predation is assumed to be a major factor underlying polymorphism. Although there is a large number of experiments showing differences in detection rates between prey of various appearances, and hence demonstrating the potential of predator visual perception in imposing selection on prey colour patterns [18], firm evidence for such selection actually resulting in variability of prey colour patterns or morph frequencies in the field is scarce, but see e.g., [19,20]. Establishing a link between phenotypic variation and rate of detection, or an association across populations between colour patterns and environmental habitat characteristics is not sufficient to conclude that visual predation has been an important driver of evolutionary shifts in animal colouration. Conclusive evidence would require demonstrating that colour pattern influences susceptibility to predation, that different colour patterns are favoured in different environments, and that predation contributes to variation in lifetime reproductive success and ultimately translates into heritable shifts of colour patterns between generations or populations [21-23].

**Figure 1:**
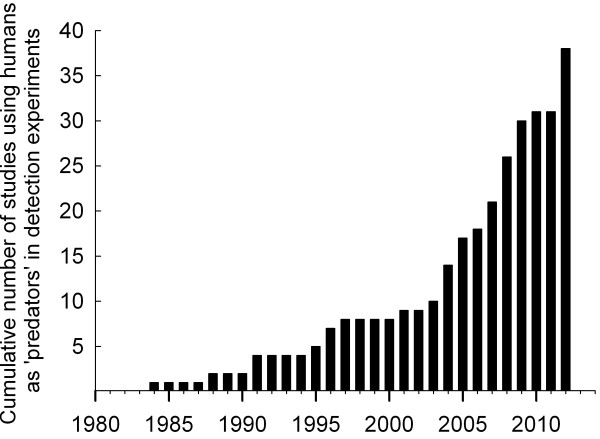
**Increase in number of published studies that have used humans as ‘predators’ in detection experiments investigating various aspects of protective coloration.** Figure shows cumulative number of studies. For list of references, see Additional file 1.

One approach to study the influence of selection for camouflage on evolution of animal colour patterns is to conduct detection experiments by presenting images of prey on paper or computer screens to human ‘predators’. This enables the investigator to control and manipulate important aspects of predator–prey interactions, and, considering how hard or unlikely it is to witness predation events in the field, this allows for levels of replication that are difficult to achieve in the wild. However, in some respects the approach lacks in realism, and will generate useful results and valid conclusions only under the assumptions that: (i) differences in rates of detection of (motionless) prey images are correlated with rates of detection and capture of live animals in the wild; and that (ii) differences in rates of detection by humans are correlated with rates of mortality imposed by natural predators. Since the seminal paper by Gendron and Staddon [24], the number of publications in which humans are used as ‘predators’ to study function and evolution of protective coloration has been rapidly increasing (Figure 1, for supporting references see Additional file 1). Previous studies have reported a striking similarity between humans and birds with regard to the ability to recognize, remember and discriminate between different conspicuous colour patterns presented against homogeneous backgrounds under artificial laboratory conditions [25,26]. Yet, no study has systematically evaluated the underlying assumptions [27], or confirmed the conjecture that rates of *detection* by humans can reliably predict and explain patterns of selection under natural conditions in the wild and evolutionary modifications of camouflaged prey colour patterns in nature.

**Figure 2:**
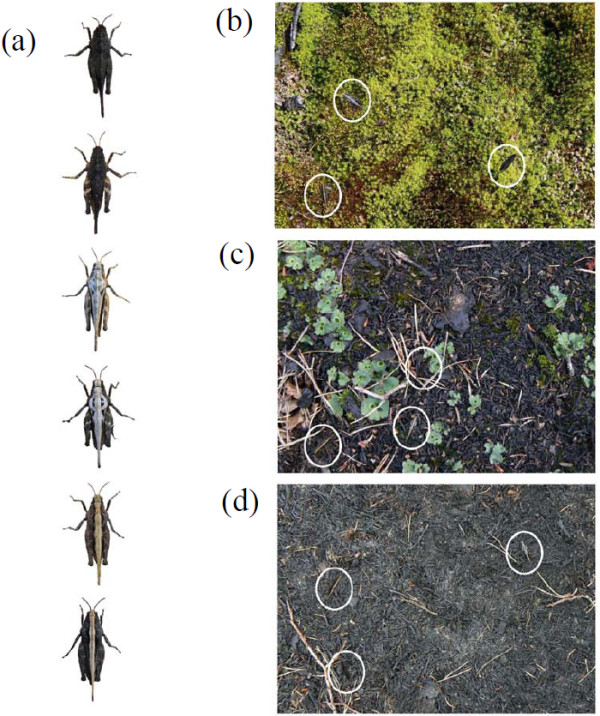
**Images of prey colour morphs and visual backgrounds used in detection experiment.***Tetrix subulata* pygmy grasshoppers representing black, grey and striped colour morphs (**a**). Images of *T. subulata* presented on photographic samples of natural backgrounds from non-burnt environment (**b**), and from post-fire environments representing 50% burnt substrate (**c**), and 100% burnt substrate (**d**). White circles denote the location of the grasshoppers. In the experiment only one grasshopper at the time was presented however in order to illustrate the differences between the colour morph on each background we present all of them in one image. Photo: E. Karpestam.

One model system in which the role of predation for the dynamics of colour polymorphism has been previously investigated is the pygmy grasshopper *Tetrix subulata* (Orthoptera: Tetrigidae) [28,29]. These are small (up to 15-mm body length, average dry body mass ca 0.07 g), diurnal, ground dwelling insects that inhabit different habitat types in biomes ranging from tropical rainforests to arctic regions of Europe, Asia and North America [7,30,31]. They range in colour from black to light grey, and some morphs are monochromatic while others have distinct patterns, consisting, for instance, of a longitudinal stripe along the median pronotum (Figure 2). Colour morphs in *T. subulata* are genetically influenced and not affected to any important degree by developmental plasticity [7] and references therein. Differences among and shifts of colour morph frequencies within populations demonstrate that pygmy grasshoppers may undergo rapid evolutionary modifications in response to environmental changes [7]. Colour morphs look similar in males and females and although morph frequencies differ somewhat between sexes within populations [29], differences in morph frequencies among populations are strongly correlated in males and females [7]. It has been confirmed that colour patterns affect the susceptibility of pygmy grasshoppers to predators [29,32,33], but whether the dynamics of the polymorphism in *T. subulata* can be explained by variation among habitat types in the selective regime imposed by visually oriented predators has not been systematically investigated. However, Tsurui *et al.*[17] found no association between differences in camouflage, as estimated by human predators, and morph frequencies in natural populations of *T. japonica.*

In the present study, our first aim is to compare the protective values of three *T. subulta* colour morphs against three types of visual backgrounds. To this end, we present human ‘predators’ with images of black, grey or striped female grasshoppers on computer screens against photographic samples of either greenish (unburnt), intermediate (ca 50% burnt) or completely burnt natural pygmy grasshopper habitats [28]. We estimate rates of detection and examine whether differences in relative protective value among morphs are consistent, or whether they change across habitat types. Our second aim is to validate the approach based on the use of humans as predators for prey presented on screen, and examine whether the results generate reliable inferences about predator–prey interactions, selection and evolution in the wild. We therefore compare rates of detection by humans of images of black, striped and grey grasshoppers presented on computer screens with independent estimates of capture and survival probabilities for the same three colour patterns obtained from a previous capture-mark-recapture study of a natural *T. subulata* population [29]. Finally, we examine whether rates of detection of the three colour morphs against different visual backgrounds are correlated with relative morph frequencies in samples of natural populations that occupied unburned, intermediate and completely burnt habitats.

## Results

### Effects of prey colour pattern and visual background on rates of detection

To test how detection was influenced by prey colour pattern and visual backgrounds, we presented human ‘predators’ with images of black, grey or striped grasshoppers on computer screens against photographic samples of either unburnt, 50% burnt or completely burnt grasshopper habitats (for images of the different prey colour morphs and visual backgrounds see Figure 2, see also Methods for details). Participants (*N* = 54) were randomly assigned to one of the 3 background types and presented with images of all 3 grasshoppers colour morphs. Colour morphs were not intermixed but presented one at a time in separate blocks. Within a block, the participant was presented with 10 habitat images in sequence, in each of which one grasshopper image of that particular morph was presented in randomized position and rotation angle. Participants were asked to search for the grasshopper image on the computer screen and to use the mouse to point and click on it. As in our previous study [28], we recorded for each prey colour morph and visual background, the number of presented prey images that were detected (correct), time to detection, the number of times that a participant failed to detect the image within 60 seconds (wrong), and the number of times the participant clicked somewhere on the screen where there was no grasshopper image (wrong).

We analyzed 1620 encounters between human predators and images of black, grey and striped grasshoppers presented on background images of unburnt, intermediate or completely burnt natural habitats. To avoid pseudo-replication, each human test participant contributed only one average value for each of the three colour morphs and for each dependent variable in the statistical analyses. Thus, for detection rate we used the percent correctly detected images out of 10 presentations. For detection time, mean values were calculated for images that were actually detected, while excluding data for presentations when the image was not detected or when the response was incorrect. Across all colour morphs and backgrounds, 73% of the presented grasshopper images were detected by the human predators. When pooled across the three backgrounds, 84% of grey, 67% of black and 66% of striped individuals were detected.

**Figure 3:**
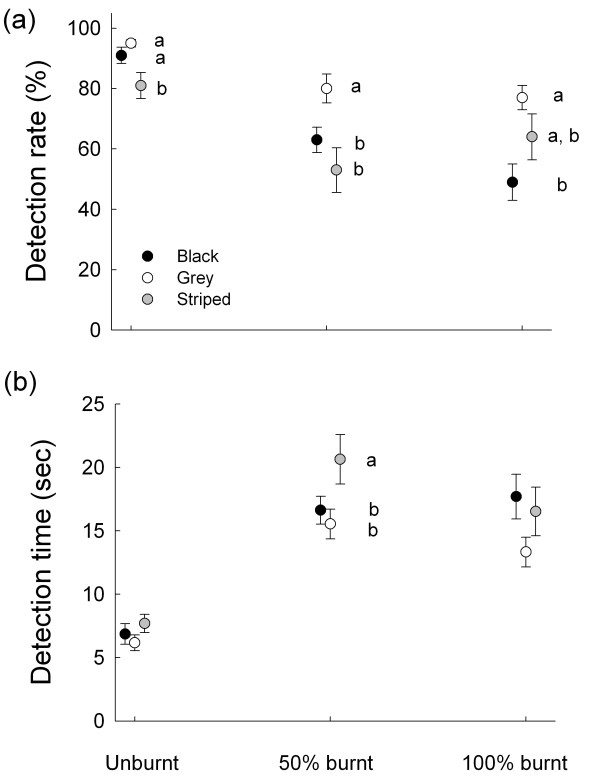
**Results from detection experiment.** Estimated rates of detection by human ‘predators’ of images of black (black circles), grey (open circles) and striped (grey circles) *Tetrix subulata* pygmy grasshoppers presented on a computer screen against images of natural unburned greenish (0% burnt), intermediate (50% burnt) and totally burned (100% burnt) visual backgrounds. Average percent detected grasshopper images (**a**). Average time to detection based on data for only those images that were detected (**b**). Figure shows mean ± s.e. Means with different letters are significantly different, as revealed by Student-Newman-Keuls a posteriori pair-wise comparisons of colour morph group means within each background.

The proportions of grasshoppers that were detected depended on colour morph (*F*_2, 102_ = 13.78, *p* < 0.001) visual background (*F*_2, 51_ = 17.43, *p* < 0.001), and on the interaction between colour morph and visual background (*F*_4, 102_ = 3.04, *p* = 0.02), the latter meaning that differences in relative rates of detection changed across visual backgrounds. Overall, grasshoppers were detected more often on unburnt backgrounds than on 50% and 100% burnt backgrounds. Results from a posteriori pair-wise comparisons obtained from separate one-way ANOVAs within each visual background (Figure 3a) show that: striped prey were detected less often than grey or black prey on unburnt backgrounds; grey prey were detected more often than black or striped prey on 50% burnt backgrounds; and in 100% burnt backgrounds the black prey were detected less often than grey prey.

Analysis of time to detection for those prey that actually were detected (i.e., including only data from trials with correct responses) revealed significant main effects of colour morph (*F*_2, 96_ = 6.71, *p* = 0.002) and background type (*F*_2, 48_ = 44.34, *p* < 0.001), but no significant interaction effect (*F*_4, 96_ = 1.60, *p* = 0.17; Figure 3b). Grasshoppers were detected faster on unburnt background than on 50% and 100% burnt backgrounds, and the results of post-hoc tests indicate that striped grasshoppers were detected more slowly than black or grey grasshoppers, but only on the 50% burned backgrounds. Overall, the results from this detection experiment show that the relative protective values (camouflage) not only differ among colour morphs, but also change across environments and microhabitats with different visual backgrounds.

### Comparisons of rates of detection on computer screens with rates of capture of live animals

**Figure 4:**
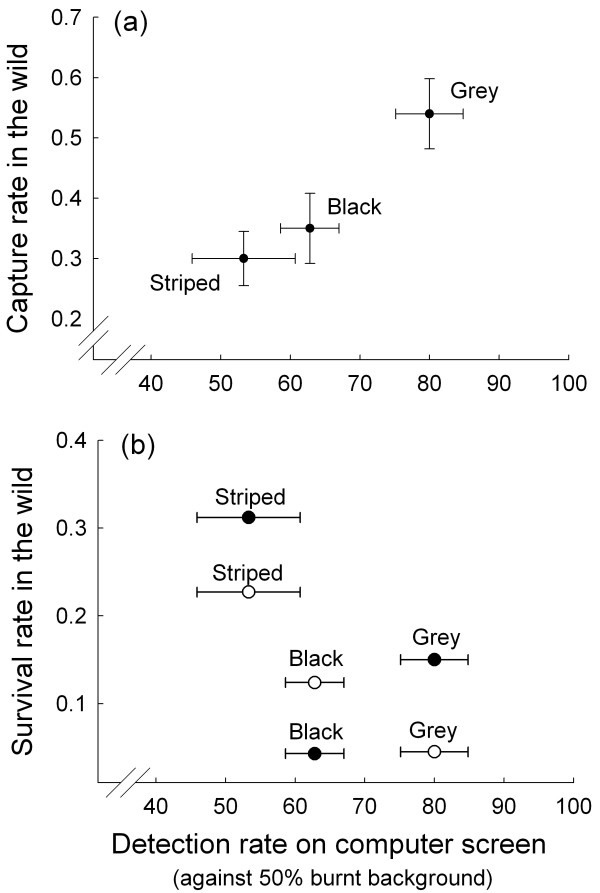
**Comparisons between detection rate, capture probability and survival in the wild.** Relationship between rate of detection by humans of images of three pygmy grasshopper colour morphs (striped, black and grey) presented against samples of visual backgrounds on a computer screen and rate of capture (**a**) and rate of survival (**b**) of free-ranging female (open symbols) and male (black symbols) live grasshoppers in the wild. Estimates of capture and total survival rate in the wild were computed from mark-recapture data. Total survival rate was computed as the product of survival probabilities for 5 separate time periods for each morph and sex. For details see Forsman and Appelqvist [29]. Figure shows mean ± s.e.

To evaluate the ecological relevance of our detection task, we compared rates of detection of black, striped and grey grasshoppers obtained in our present study with independent estimates of capture and survival probabilities of the same three colour patterns in the wild as obtained from mark-recapture data [29] (for details see Methods). Based on visual inspection of the data, differences among colour morphs in rates of detection on computer screens against 50% burnt visual background were in accordance with differences in rates of capture in a post-fire environment of free-ranging live pygmy grasshopper individuals that had been painted black, striped or grey (Figure 4a). Differences in rates of detection on computer screens corresponded also with differences in rates of survival (as estimated after controlling for the differences in capture rates, see Methods for justification) among colour patterns in female, but not in male, pygmy grasshoppers in the wild (Figure 4b). Overall, these results demonstrate that presenting human ‘predators’ with prey images on computer screens can provide reliable information on the protective value of prey colour patterns under natural conditions.

### Comparisons of rates of detection on computer screens with morph frequencies in the wild

**Figure 5:**
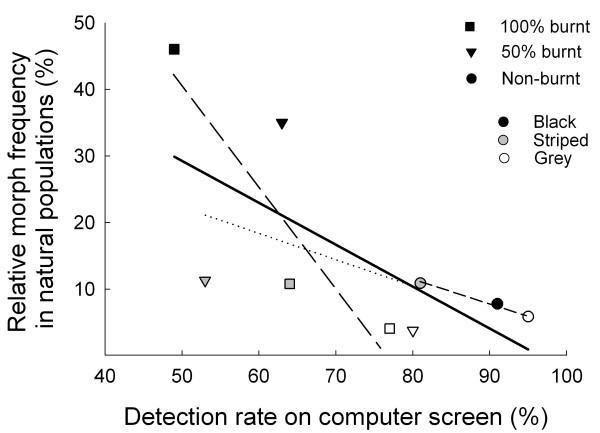
**Association between detection rates and morph frequencies in natural populations.** Relative frequencies of three pygmy grasshopper colour morphs (striped, black and grey) in natural populations from three habitat types (100% burned, 50% burned and unburnt) are negatively correlated (*r*_s_ = −0.71, *n* = 9, *p* = 0.032) with rates of detection by humans of images of grasshopper colour morphs presented against samples of visual backgrounds on a computer screen. Relative morph frequencies in the wild were estimated from data for 4,091 individuals comprising 6 samples from recently burnt (1 year after fire) habitats, 7 samples from populations in areas that had burnt 3 or 4 years prior to collection, and 14 samples from populations in unburnt areas (see Table 1). Figure shows means. Dotted and dashed lines indicate relationships among morphs within each of the three backgrounds, and the thick line indicates relationship among morphs across all backgrounds.

Finally, we evaluate whether rates of detection by humans can be used to predict and explain evolutionary modifications of relative frequencies of pygmy grasshopper colour morphs in different habitat types in the wild. To this end, we calculated the incidences of the black, grey and striped morphs based on data for 27 samples that comprised 4,091 *T. subulata* individuals collected from nonburnt areas, as well as from burnt areas of different succession stages in south-central Sweden during the period 1995 to 2012 (Table 1, see also Methods). Our data show that the incidence of the black morph was highest (45%) in recently burnt and lowest in unburnt habitats (8%; Table 1), as reported previously [7]. The relative frequencies of the striped and grey morphs were about 10% and 5%, respectively, independent of habitat type (Table 1). Overall, relative frequencies of the striped, black and grey morphs in natural populations were negatively correlated with rates of detection by humans of images of the corresponding morphs presented on computer screens against natural visual backgrounds (*r*_s_ = −0.71, *n* = 9, *p* = 0.032; Figure 5). Rates of detection were associated with relative morph frequencies both within recently burnt areas and within the unburnt habitat types. However, in the intermediate (50% burnt) habitat, the incidence of the black morph was higher than expected from its rate of detection, and the incidence of the striped morph was lower than expected from its rate of detection (Figure 5). The lower than expected incidence of the striped morph may in part represent an underestimate of the true frequency of striped grasshoppers in this habitat type, because capture probability is lower for striped than for black and grey individuals (see Methods). The grey morph which was least common in all habitat types was also detected at the highest rate in all habitat types (Figure 5). Overall, these results show that results from detection experiments with human ‘predators’ can help explain and predict diversity and micro-evolutionary shifts in animal colour patterns.

**Table 1:** Colour morph frequencies in natural pygmy grasshopper populations

**Population**	**Lat. long. coordinates**	**Burned or nonburned**	**Type of habitat**	**Year sampled**	**Years postfire**	***N***_**tot**_	***N***_**black**_	***N***_***striped***_	***N***_***grey***_
Almunge	N59º53.049’, E18º 03.813’	Burned	Clear cut (pine), managed fire, ca 20 ha	1998	1	171	68	22	17
Flyvägen	N57°00.268’, E15°16.752’	Burned	Clear cut (spruce), managed fire, ca 5 ha	2009	1	136	32	13	8
Hovmantorp	N56°47.169’, E15°10.053’	Burned	Forest (spruce), natural fire, clear cut, ca 15 ha	2009	1	114	65	15	1
Kosta	N56º51.308’, E15º35.417’	Burned	Clear cut (pine), managed fire, ca 50 ha	2006	3	221	70	17	2
=				2007	4	96	31	13	3
Nässjön	N56º53.739’, E15º18.775’	Burned	Forest (spruce), natural fire, clear cut, ca 20 ha	2008	1	38	21	4	1
Påryd	N56°36.445’,E15°53.596’	Burned	Mature forest (spruce and pine), natural fire, clear cut, ca 125 ha	2010	1	158	68	19	5
=				2012	3	86	30	12	6
Sättraby	N59º50.427’, E18º 25.229’	Burned	Clear cut (pine), managed fire, ca 10 ha	1996	3	719	305	43	51
=				1997	4	300	103	29	12
Sävsjöström	N57º00.678’, E15º26.585’	Burned	Clear cut (pine), managed fire, ca 10 ha	2004	3	347	96	41	15
Uttersberg	N59º44.638’, E15º39.439’	Burned	Clear cut (pine), managed fire, ca 10 ha	1995	1	50	29	4	1
=				1997	3	12	5	2	0
Ålem	N56°56.019’, E16°21.808’	Nonburned	Cultivated area adjacent to clear cut	2009	na	264	0	6	11
Aspelund	N56°33.226’, E16°01.468’	Nonburned	Pasture nearby small stream	2008	na	47	5	7	1
**Boden**	N65°38.969’, E22°02.507’	Nonburned	Meadow	2007	na	42	8	7	5
Bredsätra	N56°50.481’, E16°47.346’	Nonburned	Pasture nearby man-made pond	2009	na	192	1	5	17
Dammen	N59º50.298’, E18º25.275’	Nonburned	Shorelines of man-made pond	1996	na	178	28	12	18
Hägern, Boarum	N57º 25.380’, E16º15.900’	Nonburned	Pasture nearby burned area	2009	na	104	14	12	3
In Fredeln	N59º 31.707’, E19º19.225’	Nonburned	Coastal meadow, small island	1997	na	36	6	3	6
Jordtorp	N56°40.622’, E16°33.303’	Nonburned	Pasture and alkaline fen	2009	na	234	5	13	9
Läckeby, Kvilla	N56°43.953’, E15°10.546’	Nonburned	Clear cut (spruce)	2007	na	42	4	11	0
Linneryd	N56°44.496’,E15°10.868	Nonburned	Clear cut (spruce)	2007	na	30	5	1	0
Simrishamn	N55°32.986’, E14°21.116’	Nonburned	Pasture with small ponds	2012	na	26	1	9	0
Sjöbo	N55°38.917’, E13°41.644’	Nonburned	Pasture, near stream	2012	na	39	0	2	4
Sävsjö	N56°32.268’, E15°48.606’	Nonburned	Pasture and pond	2008	na	102	0	9	8
Vanserumbäck	N56°40.457’, E16°38.338’	Nonburned	Pasture	2010	na	307	3	16	10

Descriptive characteristics and frequencies of occurrence of *Tetrix subulata* colour morphs in populations sampled in burned areas of different succession stages and in nonburned areas in Sweden during the period 1995 to 2012. Total number of individuals (*N*_tot_), number of melanistic (*N*_black_), number of striped (*N*_striped_) and number of grey (*N*_grey_) individuals captured.

## Discussion

Overall, our results demonstrate that different pygmy grasshopper colour morphs vary in susceptibility to visually oriented predators, that different morphs are favoured in different visual environments, and indicate that predation may contribute to selection and spatial and temporal micro-evolutionary shifts of colour morph frequencies in natural populations. We first analyzed encounters between human ‘predators’ and images of natural black, grey and striped colour morphs of the polymorphic *Tetrix subulata* pygmy grasshoppers presented on background images of unburnt, intermediate or completely burnt natural habitats. We found that the proportion of pygmy grasshoppers that were detected and the time to detection depend both on colour patterns of the grasshoppers and on the visual properties of the background, as well as on their interaction. Our findings thus allow us to reject the null-hypothesis that detection rates of pygmy grasshoppers by visually oriented predators are independent of colour pattern. Additionally, that no single colour pattern offered superior protection against all visual backgrounds, and that the detection rate of each morph changed across backgrounds supports the notion that crypsis is context dependent [34]. The backgrounds used here were chosen such that they could be considered as different types of environments, different succession stages within temporally changing habitats, or different microhabitat patches within heterogeneous environments, and our results therefore have broad applicability.

Tsurui et al. [17] reported different detection rates by humans of *Tetrix japonica* pygmy grasshopper colour morphs against grass and sand backgrounds, but there was no indication in their study that morphs were more common in those habitat types in which they were most cryptic. Our present finding that the protective value of the black morph increases with increasing proportion burnt substrate in the visual background corroborates the results of a previous experiment that used a similar approach [28], and is consistent with the demonstration that the spatiotemporal variation in the incidence of the melanistic morph in natural *T. subulata* pygmy grasshopper populations is correlated with habitat modifications associated with succession in post-fire environments. Specifically, very high proportions of melanistic individuals in sooty environments the first year after fire are followed by rapid declines associated with recovery of vegetation and the gradual disappearance of burnt substrates [7]. That rates of detection were associated with differences in the frequencies of striped, black and grey morphs both within habitat types and across populations that occupy different types of environments (Table 1, Figure 5) adds further support to the notion [7,28] that selection for camouflage, particularly background matching is an important driver of the evolutionary dynamics of the exuberant colour polymorphism in pygmy grasshoppers.

Presumably, a high degree of background matching in one background often comes with the cost of low degree of matching in other, visually different backgrounds [34]. The consequences of such a trade-off depend on the scale of spatiotemporal variation relative to the mobility, lifespan and reproductive life history of the organism [34-38]. For instance, it has been proposed that selection in variable environments may promote the maintenance of polymorphism [35,36]. Under other conditions, environmental heterogeneity may instead select for a ‘compromise’ colour pattern that may not offer superior protection in any one habitat type but provides the best solution across a range of different habitats [34]. The striped *T. subulata* morph might represent such a compromise. It was detected at the lowest rate both in the non-burnt and in the semi-burnt treatments, and it was only against the completely burnt background that it was more easily detected than the black morph. A possible explanation for this may be that the striped pattern provides protection not only via camouflage by means of background matching, it may also impair detection or recognition by disrupting the body outline of the prey [39,40], or by closely resembling inedible objects in the environment, such as dry spruce needles, twigs or straws, i.e., via masquerade [41].

In heterogeneous environments, individuals may reduce the risk of predation further by adopting a matching habitat choice and utilize to disproportionate degrees substrate patches in the environment that offer the highest concealment [42]. Accordingly, Gillis [43] showed that green and red morphs of the grasshopper *Circotettix rabula rabula* each prefer to rest on different but matching backgrounds. Similarly, colour morphs of our current study species *T. subulata* differently utilize alternative microhabitats and surface substrates [44].

We found that the grey morph was detected at the highest rate against all three visual backgrounds. This finding is in good agreement with the observation that the frequency of grey individuals in samples from natural populations was relatively low, ca. 5%, across all three environments (Table 1), but it does not provide an explanation for the continued persistence of the grey morph. However, our three habitat types did not include all the available backgrounds influencing selection on crypsis in the pygmy grasshopper. Furthermore, visual predation is only one of many selective agents that influence the evolutionary dynamics of colour morph frequencies. Pygmy grasshopper colour morphs represent integrated phenotypes that differ in a suite of ecologically important traits such as preferred body temperatures, thermal physiology, reproductive life-history (egg and clutch size, inter-clutch interval), body size, predator avoidance behaviour, microhabitat utilization and diet e.g., [7,45,46] and references therein. Fitness differences among individuals that belong to different morphs are thus influenced not only by how difficult they are to detect, but also by selection that operates on characters and aspects of performance that are directly influenced by (such as warming up rates) or associated with colour pattern.

To use humans as ‘predators’ in detection tasks is an increasingly used approach in studies of function and evolution of protective coloration (Figure 1), but few attempts have been made to assess whether such experiments allow for reliable inferences about the influence of selection imposed by natural predators in the wild. A striking similarity has been reported between humans and birds with regard to the ability to recognize and discriminate conspicuous colour patterns presented against homogeneous backgrounds under artificial laboratory conditions [25,26]. We carried out a series of comparisons that enabled us to evaluate and validate the approach for detection of camouflaged prey in a more natural setting. First, we showed that differences in rates of detection among morphs presented on computer screens mirrored the previously reported [29] differences in rates of capture by humans of free-ranging grasshoppers in the wild. The effect of colour pattern on the relative ease with which humans can detect images of (motionless) grasshoppers in a two-dimensional representation is thus similar to the effect of colour pattern on the ability of humans to detect and capture live grasshoppers in their natural environment. Second, and more importantly, rates of detection on computer screens were correlated with estimates of survival (adjusted for differences in capture probabilities) of striped, black and grey female (but not male) *T. subulata* in the wild, thus demonstrating that detection rates by humans may reliably predict selection imposed by natural visual predators, such as birds e.g., [47]. That colour pattern differently influences survival of male and female pygmy grasshoppers in the wild can be attributed to females being larger, utilizing different microhabitats, and being more active (longer average daily movement distances) than males [29], since the protective value of a given colour pattern may depend on body size [48], behaviours and movement patterns [32], and visual backgrounds [28]; this study. In the current study, however, body size and behaviours did not differ between images that represented different colour morphs and body size corresponded to that of large females. Third and finally, detection rates of grasshopper images were correlated with relative frequencies of striped, black and grey morphs in 27 samples of more than 4,000 *T. subulata* individuals collected from natural populations that occupied the same habitat types that were used as visual backgrounds in the detection experiment. These findings implicate visual predation as an important driver of evolutionary modifications of colour polymorphism in pygmy grasshoppers, and demonstrate that, in our system, using humans as ‘predators’ in detection experiments contributes reliable and relevant information that enhances our understanding of natural selection and evolution.

## Conclusions

To investigate whether selection imposed by visual predators influence evolutionary modifications of animal colour patterns we presented human ‘predators’ with images of grey, black and striped *T. subulata* pygmy grasshoppers on a computer screen against photographic samples of natural backgrounds representing unburnt, intermediate or completely burnt environments. Using this approach, we demonstrated that the proportion of grasshoppers that were detected and time to detection depended on the colour pattern of the prey and on the type of visual background, providing clear evidence that crypsis is context dependent. We also showed that differences among morphs in rates of detection on computer screens mirrored previously reported morph-specific differences in survival of free-ranging grasshoppers in the wild, and were correlated with relative frequencies of striped, black and grey morphs in natural populations. Our findings implicate visual predation as an important driver of evolutionary modifications of colour polymorphism in pygmy grasshoppers, and provide clear evidence that, at least in this system, see also [25], using humans as ‘predators’ in detection experiments may offer reliable information on the protective values of colour patterns and of natural selection and evolution in the wild.

## Methods

The general design of the detection task experiment followed a recent study [28], except that we used three different prey colour morphs rather than one, and three types of visual backgrounds rather than five.

### Photographic sampling of visual backgrounds and grasshopper colour morphs

Photographs of *T. subulata* habitats were taken in summer 2010 with a digital compact camera (Panasonic Lumix DMC-TZ7, 35 mm focal length equivalent) using ‘macro’ mode at a vertical distance of approximately 30 cm straight from above. Pictures were taken in several heterogeneous environments at three locations in south east Sweden that had been previously heavily influenced by natural or managed forest fires, at different stages of recovery, and that were surrounded by healthy unburnt forest. The three sites were Smedjevik (N56°59.335, E16°5.743, burnt 2010), Åsjön (N56°58.685, E16°4.6213, burnt 2010) and Påryd (N56°36.445, E15°53.596, burnt 2009). We photographed patches that represented three visual backgrounds and habitat types (Figure 2). Completely burnt patches with a surface covered by charcoal and sooty material such as branches, twigs, needles and cones (100% burnt). Intermediate patches that contained both burnt and un-burnt surface substrates (50% burnt). Un-burnt and recovered patches characterized by green vegetation, including different types of mosses (un-burnt).

Adult *T. subulata* female grasshoppers were collected at the same sites where the background pictures were taken. We collected individuals that belonged to the black, grey, or striped colour morph and brought them the lab for photographing. These morphs where chosen because they are distinct from each other (Figure 2), because their frequencies vary both among populations and over time within populations [7], and because estimates of capture and survival probabilities for these morphs are available from a previous study [29]. Grasshoppers were individually photographed with the same digital compact camera using ‘macro’ mode approximately 7 cm (minimum distance for macro-mode) from above on brownish cardboard in a shaded area under natural daylight summer conditions outdoors for details see [28]. Overall, our photographs of backgrounds included variable light conditions. Therefore, in the experimental presentations grasshoppers may have appeared somewhat different on different backgrounds, and on the same type of background with different light conditions, corresponding to heterogeneous natural conditions in the wild.

We used the software Adobe Photoshop CS4 for image processing. Grasshopper images were cut from their original background and saved on transparent background in order to enable implementation in background images [28]. All grasshoppers were measured for body length (pronotum) in the photos and rescaled to the identical size of 15 mm on the screen (the natural size of large females), to avoid any bias towards easier detection of larger individuals. During the training sessions and experimental presentations (see below) images were presented on a 15’ computer screen (Fujitsu Lifebook e series) in a dark room.

### Human predators

Fifty-four people (27 males and 27 females) were randomly, but with similar proportions of males and females, assigned to one of the three background groups, giving 18 participants in each treatment. Participants ranged in age from 17–63 (mean = 32, s.d. = 11.4) years and there were no age differences between participants assigned to the 3 different types of visual backgrounds (*F*_2,51_ = 0.031, *p* = 0.73). All participants (students and personnel at Linnaeus University) had normal or corrected to normal vision. In contrast to humans, some insectivorous predators can detect UV light, but Tsurui et al. [17] measured very low reflectance of UV from *Tetrix* grasshoppers.

Prior to the experimental presentations, each participant received a brief verbal instruction of the task from EK. To familiarize the participants with the experimental setup and with the grasshoppers’ size, shape and colour, we next presented a grasshopper image on white background on the computer screen for 20 seconds. This was followed by two training sessions, in which the grasshopper image was implanted first on an image of a non-burnt background, and then on an image showing a completely burnt background. All participants received identical instructions and training sessions. After the practice block, each participant was left alone in the room to perform 10 experimental trials of the detection task (see below). All participants were presented with 10 images each of all three colour morphs (yielding a total of 30 experimental trials per subject), but in separate experimental sessions in a balanced design (see below), and with a separate training session to familiarize them with the specific colour morph before each experimental session. The instruction, training sessions and experiments were all conducted in a dark room to avoid reflections on the computer screen. We conducted our study abiding by the Swedish legislation and ethical regulations for experimental research. For this type of experiment (a detection task in which images are presented to humans) an ethical permission is not needed.

### Programming and detection task

For presentation of prey images in the detection task experiment, we produced a purpose-written computer programme using MATLAB 2011 software [28]. To mimic the natural situation it is necessary to take into consideration small scale heterogeneity of visual backgrounds within each habitat type. In the experiment, we therefore used 7 background images for each of the 3 habitat types (unburnt, 50% burnt and 100% burnt). Similarly, we selected images of 7 grasshopper individuals for each of the three colour morphs (black, striped or grey), in order to increase the generality of our results and to simulate the situation in wild populations, in which there is subtle variation in details of the patterning and hue also within the different morph categories (Figure 2).

Participants (*N* = 54) were strategically randomized, assigned to one of the 3 background types and presented with all 3 colour morphs. Thus, we used 18 participants for each background type. Colour morphs were not intermixed but presented one at a time in separate blocks. Within a block, the participant was presented with 10 habitat images in sequence, into each of which one of the 7 grasshopper images of that particular morph was implanted. Each participant thus carried out 3 experimental blocks and contributed a total of 30 separate trials. We used a fully balanced design. For each background type, 6 participants started with the black grasshoppers, 6 others started with the striped grasshoppers, and the remaining 6 started with the grey grasshoppers. The sequence of morphs in the middle and last blocks were interchanged in similar way.

Within each block, and for each presentation, the program randomly combined one background image and one grasshopper image from each of the seven grasshoppers and backgrounds available. The grasshopper was implanted in randomized position and rotation angle (0-360°) on the background image (Figure 2). The subject was then asked to search for the grasshopper and use the mouse to point and click on it. After the click, the program recorded the result (‘correct’ if on the grasshopper, else ‘wrong’) and the time to detection, and a new combination of images was presented. If a test subject did not click the mouse within 60 seconds from the beginning of a presentation, the result of the trial was recorded as ‘wrong’ and detection time as 60 sec, and a new combination of images was presented.

### Statistical analyses of data from the detection experiment

In the analyses of detection rate, we calculated the percent correctly detected images, out of 10 presentations, for each subject and colour morph. In the test for differences in time to detection, individual mean values were calculated based on data for grasshopper images that actually were detected, while data for those presentations when the image was not detected or when the response was incorrect were excluded.

We performed separate analyses for each of the two dependent variables. Data on proportion detected images were arcsine transformed for normality and homogeneity of variances, and data on time to detection were square root transformed prior to analyses. To test for effects of colour morph, visual background and their interaction on proportion detected individuals and on the time to actual detection, we used two-way repeated measure ANOVAs, treating colour morph as a within-participant variable. Because the colour morph by visual background interaction was significant, we performed a separate one-way repeated measure ANOVA for each of the three visual backgrounds, with colour as a within-participant variable, and used the SNK method for a posteriori pair-wise comparisons of colour morph group means within each background.

### Rates of capture by humans and survival of free-ranging grasshoppers in the wild

We obtained independent estimates of capture rates and survival probabilities from a previous experiment by Forsman and Appelqvist [29] who carried out a capture-mark-recapture study of free-ranging *T. subulata* that were painted black, striped or grey. Their study was performed between 8^th^ of May and the 26^th^ of June, 1996, in a natural population inhabiting a clear cut area (previous pine forest) that had been intentionally burnt for conservation purposes 3 years earlier, and the environment corresponded to the 50% burnt visual background treatment used in our present detection experiment. Recapture histories for 442 experimentally manipulated individuals were used to obtain independent estimates of both *capture rates* (by humans) and *survival rates* for the different paint treatments [29]. The main virtue of computing independent estimates of rates of capture and survival is that the differences among colour morphs (if any) in rates of capture by humans can be statistically controlled for when estimating and comparing rates of survival. Otherwise, recapture rates reflect the combined effects of differences among morphs in capture rates and ‘true’ survival. Capture rates differed between colour morphs but were independent of sex. Survival probabilities differed both between morphs, sexes and time periods. Striped individuals survived best in both males and females; whereas black females survived better than grey females, and grey males survived better than black males. To obtain point estimates of relative survival throughout the course of the study, we calculated the product of survival probabilities for each of the 5 time periods for each morph and sex [29].

### Estimating relative morph frequencies in natural populations

We estimated relative frequencies of the black, grey and striped pygmy grasshopper colour morphs in different habitat types in the wild based on data for 27 samples that comprised 4,091 *T. subulata* individuals collected from nonburnt areas, as well as from burnt areas of different succession stages in southeast Sweden during the period 1995 to 2012 (Table 1). Grasshoppers were collected by hand, usually in spring and early summer, while walking through different areas during days with weather conditions suitable for grasshopper activities [7]. *T. subulata* does not have a uniform or random spatial distribution, and we therefore initially searched the entire areas and then concentrated our search and capture effort to those parts and microhabitats (i.e., humid bare soil, areas covered by mosses) that offered suitable conditions for pygmy grasshoppers. Because *T. subulata* predominantly move around on the ground surface and rarely climb vegetation they are difficult to capture with a bag net, and we therefore acted like visual predators and captured individuals that we could see [7,29]. Since striped individuals are more difficult to detect and capture than black or grey individuals in burned environments [29] (see also Figure 3), the incidence of the striped morph may be underestimated in our samples from the burned environments described below. Such a bias would have a conservative influence on our results, making it more difficult to detect an association between detectability and relative morph frequencies in the wild. Data for non-burned areas consisted of 14 samples (1,643 individuals) from populations in diverse habitat types, such as pastures, clear cut fields and coastal meadows. Data for habitats representing the 50% burnt background consisted of 7 samples (1,781 individuals) from populations in areas that had been ravaged by natural or managed fires 3 or 4 years prior to collection. Finally, data for habitats representing the 100% burnt visual background, consisted of 6 samples (667 individuals) from recently burnt (1 year after fire) habitats (Table 1). We also have samples collected two years after fire events [7], but they were excluded from this study to ensure that our estimated morph frequencies represented distinct succession stages. As reported previously, differences in morph frequencies among samples from different populations and years are strongly correlated in males and females [7], and we therefore pooled data for the two sexes in the statistical analyses of data. We used Spearman rank correlation analysis to test if relative morph frequencies in natural populations were negatively correlated with rates of detection by humans of images on computer screens.

## Abbreviations

ANOVA: Analysis of variance; rs: Spearman rank correlation coefficient; SE: Standard error; SEC: Seconds.

## Competing interests

The authors declare that they have no competing interests.

## Authors’ contributions

All authors designed the detection experiment; EK photographed animals and backgrounds, wrote the presentation program, and undertook the detection experiment; EK and AF performed the statistics. All authors contributed to the writing and read and approved the final manuscript.

## Supplementary Material

Additional file 1 **Supporting references for Figure** **1.** List of studies in which images of (artificial) prey on paper or computer screens have been presented to human ‘predators’ to investigate various aspects of protective coloration. Click here for file
