# Supplementary material for: Detection experiments with humans implicate visual predation as a driver of colour polymorphism dynamics in pygmy grasshoppers
Source: BMC Ecol. 2013 May 2;13:17. doi: 10.1186/1472-6785-13-17 (PMC3648452; doi:10.1186/1472-6785-13-17)
Supplement: Additional file 1 — Supporting references for Figure 1. List of studies in which images of (artificial) prey on paper or computer screens have been presented to human ‘predators’ to investigate various aspects of protective coloration. [file 1472-6785-13-17-S1.pdf]

Additional file 1: Additional supporting references for Figure 1.

Detection experiments with humans implicate visual predation as a driver of colour polymorphism dynamics in pygmy grasshoppers

Einat Karpestam, Sami Merilaita, Anders Forsman[1]

[1] Author for correspondence E-mail: anders.forsman@lnu.se

List of studies in which images of (artificial) prey on paper or computer screens have been presented to human 'predators' to investigate various aspects of protective coloration.

Beatty CD, Bain RS, Sherratt TN (2005) The evolution of aggregation in profitable and unprofitable prey. Anim Behav 70:199-208

Beatty CD, Beirinckx K, Sherratt TN (2004) The evolution of mullerian mimicry in multispecies communities. Nature 431:63-67

Bohlin T, Gamberale-Stille G, Merilaita S, Exnerova A, Stys P, Tullberg BS (2012) The detectability of the colour pattern in the aposematic firebug, *Pyrrhocoris apterus*: an image-based experiment with human 'predators'. Biol J Linn Soc 105:806-816

Bohlin T, Tullberg BS, Merilaita S (2008) The effect of signal appearance and distance on detection risk in an aposematic butterfly larva (*Parnassius apollo*). Anim Behav 76:577-584

Cook LM, Kenyon G (1991) Frequency-dependent selection with background heterogeneity. Heredity 66:67-73

Cooper WE, Jr., Caldwell JP, Vitt LJ (2008) Effective crypsis and its maintenance by immobility in Craugastor frogs. Copeia 527-532

Cuthill IC, Szekely A (2009) Coincident disruptive coloration. Phil Trans R Soc B 364:489-496

Fraser S, Callahan A, Klassen D, Sherratt TN (2007) Empirical tests of the role of disruptive coloration in reducing detectability. Proc R Soc B 274:1325-1331

Gendron RP, Staddon JER (1984) A laboratory simulation of foraging behavior - the effect of search rate on the probability of detecting prey. Am Nat 124:407-415

Glanville PW, Allen JA (1997) Protective polymorphism in populations of computer-simulated moth-like prey. Oikos 80:565-571

Jackson AL, Brown S, Sherratt TN, Ruxton GD (2005) The effects of group size, shape and composition on ease of detection of cryptic prey. Behaviour 142:811-826

Jones KA, Jackson AL, Ruxton GD (2011) Prey jitters; protean behaviour in grouped prey. Behav Ecol 22:831-836

Karpestam E, Merilaita S, Forsman A (2012) Reduced predation risk for melanistic pygmy grasshoppers in post-fire environments. Ecology and Evolution 2:2204-2212

Knill R, Allen JA (1995) Does polymorphism protect - an experiment with human predators. Ethology 99:127-138

Kreisinger J, Albrecht T (2008) Nest protection in mallards *Anas platyrhynchos*: untangling the role of crypsis and parental behaviour. Funct Ecol 22:872-879

McGuire L, Van Gossum H, Beirinckx K, Sherratt TN (2006) An empirical test of signal detection theory as it applies to Batesian mimicry. Behav Processes 73:299-307

Penney HD, Hassall C, Skevington JH, Abbott KR, Sherratt TN, (2012) A comparative analysis of the evolution of imperfect mimicry. Nature 483, 461-464.

Reynolds C (2011) Interactive evolution of camouflage. Artificial Life 17:123-136

Ruxton GD, Jackson AL, Tosh CR (2007) Confusion of predators does not rely on specialist coordinated behavior. Behav Ecol 18:590-596

Sherratt TN, Beatty CD (2003) The evolution of warning signals as reliable indicators of prey defense. Am Nat 162:377-389

Sherratt TN, Pollitt D, Wilkinson DM (2007) The evolution of crypsis in replicating populations of web-based prey. Oikos 116:449-460

Sherratt TN, Rashed A, Beatty CD (2004) The evolution of locomotory behavior in profitable and unprofitable simulated prey. Oecologia 138:143-150

Stevens M, Searle WTL, Seymour JE, Marshall KLA, Ruxton GD (2011) Motion dazzle and camouflage as distinct anti-predator defenses. BMC Biology 9:81

Stevens M, Yule DH, Ruxton GD (2008) Dazzle coloration and prey movement. Proc R Soc B 275:2639-2643

Summers K, Clough ME (2001) The evolution of coloration and toxicity in the poison frog family (Dendrobatidae). Proc Natl Acad Sci USA 98:6227-6232

Todd PA, Lee JH, Chou LM (2009) Polymorphism and crypsis in the boring giant clam (*Tridacna crocea*): potential strategies against visual predators. Hydrobiologia 635:37-43

Tosh CR, Krause J, Ruxton GD (2009) Basic features, conjunctive searches, and the confusion effect in predator-prey interactions. Behav Ecol Sociobiol 63:473-475

Tosh CR, Ruxton GD, Krause J, Franks DW (2011) Experiments with humans indicate that decision accuracy drives the evolution of niche width. Proc R Soc B 278:3504-3509

Tsurui K, Honma A, Nishida T (2010) Camouflage effects of various colour-marking morphs against different microhabitat backgrounds in a polymorphic pygmy grasshopper *Tetrix japonica*. PLoS One 5: e11446

Tsurui K, Honma A, Nishida T (2012) Size-dependent predation risk partly explains the sex-related marking polymorphism in the sexually size-dimorphic pygmy grasshopper *Tetrix japonica*. Entomological Science. DOI: 10.1111/j.1479-8298.2012.00543.x

Tucker GM, Allen JA (1988) Apostatic selection by humans searching for computer-generated images on a color monitor. Heredity 60:329-334

Tucker GM, Allen JA (1991) Selection by humans searching for computer-generated prey images - the effect of prey density. Biol J Linn Soc 44:169-174

Tullberg BS, Gamberale-Stille G, Bohlin T, Merilaita S (2008) Seasonal ontogenetic colour plasticity in the adult striated shieldbug *Graphosoma lineatum* (Heteroptera) and its effect on detectability. Behav Ecol Sociobiol 62:1389-1396

Tullberg BS, Merilaita S, Wiklund C (2005) Aposematism and crypsis combined as a result of distance dependence: functional versatility of the colour pattern in the swallowtail butterfly larva. Proceedings of the Royal Society B 272:1315-1321

Van Gossum H, Adriaens T, Dumont H, Stoks R (2004a) Sex- and morph-specific predation risk: Colour or behaviour dependency? Euro J Entomol 101:373-377

Van Gossum H, Stoks R, De Bruyn L (2004b) Conspicuous body coloration and predation risk in damselflies : are andromorphs easier to detect than gynomorphs? Belg J Zool 134:37-40

Webster RJ, Callahan A, Godin J-GJ, Sherratt TN (2009) Behaviourally mediated crypsis in two nocturnal moths with contrasting appearance. Phil Trans R Soc B 364:503-510
